# P-1317. Beyond Clinical Factors: Examining the Association between Socioeconomic Status and Severe Mpox Disease in NYC

**DOI:** 10.1093/ofid/ofae631.1497

**Published:** 2025-01-29

**Authors:** Ofole Mgbako, Yusra Shah, Anthony J Lo Piccolo, Kathryn Jano, Alvin M Ho, Madeline DiLorenzo, Dorothy Knutsen, Dana Mazo, Joyce C Pressley, Jason Felder, Dustin Duncan

**Affiliations:** NYU Grossman School of Medicine, Brooklyn, New York; New York University, Manhattan, New York; NYU Langone Health, New York, New York; NYU Grossman School of Medicine, Brooklyn, New York; NYU Langone Health, New York, New York; NYU Langone Health, New York, New York; NYU Langone, New York, New York; New York University, Manhattan, New York; Columbia University, New York, New York; NYU Langone, New York, New York; Columbia University, New York, New York

## Abstract

**Background:**

The NYC 2022-23 mpox outbreak predominantly affected sexual minority men with notable racial/ethnic disparities. Clinical predictors of severe mpox disease (e.g., poorly controlled HIV) have been established, but little is known about the socioeconomic predictors of severe disease.

**Figure:**
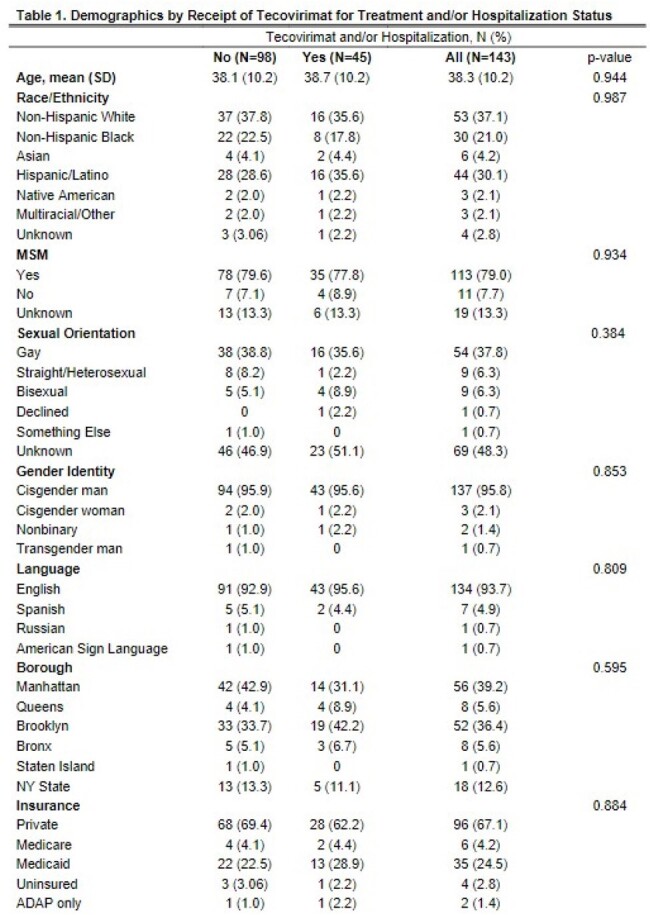

**Methods:**

Patients age ≥ 18 with a confirmed mpox diagnosis from 5/1/2022-12/31/2023 (N=143) were identified through the electronic medical record at an academic medical center in NYC. Low socioeconomic status (SES) (N=41) was defined as being uninsured (N=4), on Medicaid (N=35), or in the AIDS Drug Assistance Program (ADAP) (N=2). We characterized patients by demographics and clinical characteristics and compared those who received tecovirimat for treatment and/or who were hospitalized with those who were not. We operationalized disease severity using the newly validated mpox severity score (mpox-SS). We then examined associations between SES and mpox-SS, controlling for age, race/ethnicity, mental health, substance use, high risk status (e.g., HIV with CD4 < 350cells/mm3), prior mpox vaccination, and STD diagnosis.

**Figure:**
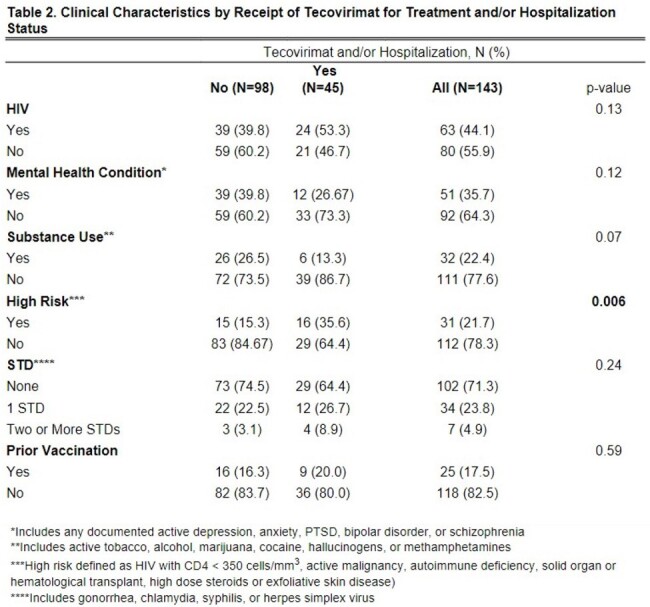

**Results:**

Demographics (Table 1) and clinical characteristics (Table 2) were examined by tecovirimat and/or hospitalization. Over 67% of patients were privately insured. About 44% had HIV, 17% had prior mpox vaccination, and 22% were high risk. About 26% received tecovirimat, 15% were hospitalized inpatient, while 3% were admitted to ICU. There were 135 patients with clinical data to calculate the mpox-SS. The mean (SD) mpox-SS was 6.85 (3.36) (Figure 1). In univariate analysis, low SES was significantly associated with more severe mpox-SS (p=0.02) (Table 3) and remained significant in the model (p=0.03) controlling for race/ethnicity, high risk status, STD, or prior vaccination. High risk status was associated with higher mpox-SS and prior vaccination was associated with lower mpox-SS.

**Figure:**
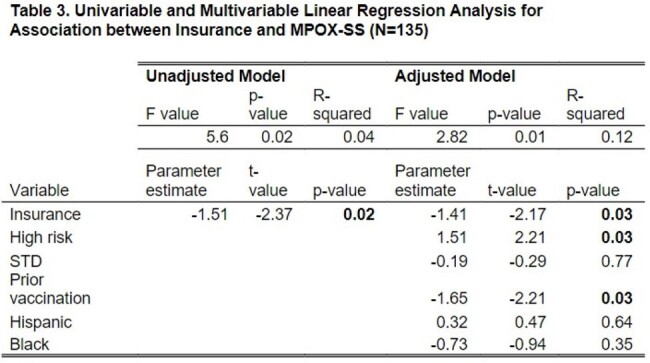

**Conclusion:**

Using the mpox-SS, low SES was significantly associated with higher mpox severity in this diverse cohort of cisgender sexual minority men in NYC, likely reflecting delays in access to care or other inequities. Further studies are needed to assess the relationship between SES and severe mpox in order to understand factors beyond insurance that lead to disparate clinical mpox outcomes.

**Figure:**
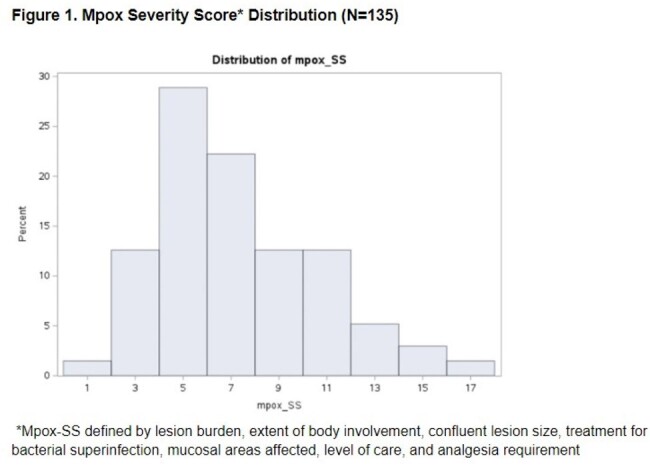

**Disclosures:**

**Ofole Mgbako, MD MS**, Gilead: Advisor/Consultant **Madeline DiLorenzo, MD**, Abbvie: Stocks/Bonds (Public Company)|Agilent Technologies: Stocks/Bonds (Public Company)|Amgen: Stocks/Bonds (Public Company)|Becton Dickinson: Stocks/Bonds (Public Company)|Biogen: Stocks/Bonds (Public Company)|BioTechne Corp: Stocks/Bonds (Public Company)|Bristol Myers Squibb: Stocks/Bonds (Public Company)|Cardinal Health: Stocks/Bonds (Public Company)|Centene Corp: Stocks/Bonds (Public Company)|CVS Health: Stocks/Bonds (Public Company)|Davita Inc: Stocks/Bonds (Public Company)|Ecolab: Stocks/Bonds (Public Company)|Elevance Health: Stocks/Bonds (Public Company)|Gilead Sciences Inc.: Stocks/Bonds (Public Company)|Glaxo Smith Kline: Stocks/Bonds (Public Company)|HCA Healthcare Inc: Stocks/Bonds (Public Company)|Healthpeak Properties: Stocks/Bonds (Public Company)|Henry Schein: Stocks/Bonds (Public Company)|Hologic Inc: Stocks/Bonds (Public Company)|Humana Inc: Stocks/Bonds (Public Company)|Idexx Laboratories: Stocks/Bonds (Public Company)|Jazz Pharmaceuticals: Stocks/Bonds (Public Company)|Labcorp: Stocks/Bonds (Public Company)|Merck and Company: Stocks/Bonds (Public Company)|Quest Diagnostics Inc: Stocks/Bonds (Public Company)|Resmed Inc: Stocks/Bonds (Public Company)|ThermoFisher Scientific: Stocks/Bonds (Public Company)|Vertex Pharmaceuticals: Stocks/Bonds (Public Company)|West Pharmaceuticals: Stocks/Bonds (Public Company)

